# Best practice in post‐diagnosis care for people with dementia through Recovery Colleges (DiSCOVERY‐Post‐Diagnostic Dementia Support within the ReCOVERY College Model)

**DOI:** 10.1002/alz70858_098844

**Published:** 2025-12-25

**Authors:** Chris Fox, Juniper West, Geoff Wong, Emma Wolverson, Fiona Poland, Melanie Handley, Linda Birt, Esme Moniz‐Cook, Corinna Hackmann, Bonnie Teague

**Affiliations:** ^1^ University of Exeter, Exeter, Devon, United Kingdom; ^2^ NIHR Healthtech research centre, Exeter, United Kingdom; ^3^ university of east anglia, Norwich, United Kingdom; ^4^ Norfolk and Suffolk NHS Foundation Trust, Norwich, United Kingdom; ^5^ Oxford University, Oxford, United Kingdom; ^6^ The University of West London, London, England, United Kingdom; ^7^ University of East Anglia, Norwich, United Kingdom; ^8^ University of Hertfordshire, Hatfield, United Kingdom; ^9^ University of Leicester, Leicester, United Kingdom; ^10^ Institute of Rehabilitation, Dementia Applied Research Centre, University of Hull, Hull, United Kingdom; ^11^ NSFT, Norwich, Norfolk, United Kingdom; ^12^ NSFT, Norwich, United Kingdom

## Abstract

**Background:**

Post‐diagnostic support in dementia is highly variable often focusing on attempting to retain cognitive skills or information giving, rather than actively embracing concepts which may enhance personal ‘recovery’ from receiving a dementia diagnosis. Recovery colleges are increasingly being viewed as a viable method of delivering post‐diagnostic support

Recovery College courses ‐ usually co‐produced with people with a dementia‐diagnosis, may help people to adjust to their diagnosis, by maintaining their social connections, challenging stigmatising stereotypes and actively engaging in shaping their post‐diagnosis experiences, thus enabling them to live positively with disability. Little is known about the extent, range of content and effects on experience and sustaining connections, of those who attend Recovery College courses for people with dementia. For people with dementia who attend Recovery College courses, little is known about the range of content, effects on experiences and in sustaining connections. This study aimed to examine what works, how and for whom, in what contexts.

**Method:**

A realist evaluation, set in four English mental health services collected interview data, ethnographic observations and documentary evidence from five courses.

**Results:**

Observation data from five courses (case studies) and interviews with 13 tutors (including 3 people with dementia) and 32 course attendees (8 people with dementia) were analysed. Realist evaluation methodology led to the development of four overarching Context‐Mechanism‐Outcome Configurations (26 granular CMOCs) related to staging a course. Shared commitment to co‐production values across staff and peer tutors was essential to setting‐up and running courses successfully; people known to attendees facilitated attendance at courses; course delivery foregrounded principles of problem‐solving, reducing stigma and moving forward with hope; course evaluation was challenging to achieve.

**Conclusion:**

DiSCOVERY utilised mixed methodology to develop a blueprint for patients, family supporters, staff and providers to set up and sustain Recovery College dementia courses, which is coproduced, peer‐led education focused on living with dementia positively and with hope, based on learning from five national course case studies. This learning can provide a framework to develop recovery colleges for dementia internationally.

